# Coarse-to-Fine Construction for High-Resolution Representation in Visual Working Memory

**DOI:** 10.1371/journal.pone.0057913

**Published:** 2013-02-28

**Authors:** Zaifeng Gao, Xiaowei Ding, Tong Yang, Junying Liang, Rende Shui

**Affiliations:** Department of Psychology and Behavioral Sciences, Zhejiang University, Hangzhou, China; University of Regensburg, Germany

## Abstract

**Background:**

This study explored whether the high-resolution representations created by visual working memory (VWM) are constructed in a coarse-to-fine or all-or-none manner. The coarse-to-fine hypothesis suggests that coarse information precedes detailed information in entering VWM and that its resolution increases along with the processing time of the memory array, whereas the all-or-none hypothesis claims that either both enter into VWM simultaneously, or neither does.

**Methodology/Principal Findings:**

We tested the two hypotheses by asking participants to remember two or four complex objects. An ERP component, contralateral delay activity (CDA), was used as the neural marker. CDA is higher for four objects than for two objects when coarse information is primarily extracted; yet, this CDA difference vanishes when detailed information is encoded. Experiment 1 manipulated the comparison difficulty of the task under a 500-ms exposure time to determine a condition in which the detailed information was maintained. No CDA difference was found between two and four objects, even in an easy-comparison condition. Thus, Experiment 2 manipulated the memory array’s exposure time under the easy-comparison condition and found a significant CDA difference at 100 ms while replicating Experiment 1′s results at 500 ms. In Experiment 3, the 500-ms memory array was blurred to block the detailed information; this manipulation reestablished a significant CDA difference.

**Conclusions/Significance:**

These findings suggest that the creation of high-resolution representations in VWM is a coarse-to-fine process.

## Introduction

By providing online storage for a limited amount of visual information transferred from visual perception, visual working memory (VWM) plays a critical role in our dynamic and coherent visual experience [1], [2], [3] and many other high-level cognitive activities [4], [5], [6]. In recent years, a growing number of studies have attempted to elucidate the processing mechanisms of VWM, such as representation maintenance [7], [8], [9], [10], [11], consolidation [12], retrieval and comparison [13], [14], [15], [16]. However, how the representations in VWM are constructed based on the perceptual input remains largely unclear. Here we explored this question by asking whether VWM representations containing detailed (high-resolution) information are constructed in a gradual, coarse-to-fine manner or a discrete, all-or-none manner. This issue is not only important for VWM since it encodes and stores detailed information in daily life [17], [18], but will also have great implications on the processing mechanisms of high-level cognition in general.

Coarse-to-fine processing is important in our vision system. Perceptual studies have convincingly demonstrated that coarse information precedes detailed information in entering the perceptual system [19], [20], [21], [22], [23], [24], [25], [26]. The recent neural object file theory suggests that this cascade processing also exists in VWM [18]. Proto-objects are created in VWM on the basis of input consisting of coarse information to guide further processing, such that processing time and resources are considerably saved. Moreover, a previous study on encoding visual scenes into VWM also revealed coarse-to-fine processing of scenes containing various types of visual information [17]. However, to our knowledge, only one recent study has explicitly tested the hypothesis that object representations are constructed in a coarse-to-fine manner in VWM. Using human faces as materials, Gao and Bentin [27] demonstrated that low spatial frequency, which conveys coarse information, precedes high spatial frequency, which conveys detailed information, in entering VWM. Considering the processing specialty of human faces in visual system [28], [29] and that behavioral performance could not track the storage phase of VWM directly, more empirical evidence is needed to test this coarse-to-fine hypothesis.

On the other hand, seemingly supporting the specialization of VWM in constructing representations of human faces, certain evidence implies that VWM representation proceeds in an all-or-none encoding manner [30], [31]. This hypothesis suggests that either all or none of the task-relevant visual information contained in an object is encoded into VWM in an unordered fashion. A recent study provided direct evidence for this manner of encoding [31]: in a VWM experiment, three simple colors were displayed for 100 ms, and 10 ms or 240 ms after their offset, they were backward-masked to examine the encoding mechanism of color information. In contrast to the prediction of the gradual encoding hypothesis that the resolution of representations should be affected by their consolidation time, the resolution of stored color in VWM was constant. Only the probability of colors being stored in VWM was affected. However, this finding may be restricted to coarse information, since distinct colors and short encoding and consolidation times were used (see also the discussion in [32]). Therefore, it remains unclear whether or not the all-or-none processing manner also applies to common complex objects containing detailed information.

To this end, we investigated the construction process of high-resolution representations in VWM. Instead of relying on behavioral performance, which is affected by many other factors and thereby could not reflect the maintenance phase directly [33], [34], we adopted the ERP component of contralateral delay activity (CDA) as the main index of interest, because it is an online and objective index of object information stored in VWM [35], [36], [37], [38]. More importantly, CDA is sensitive to the resolution of the memorized objects, as CDA under exposure to two and four objects differs according to the level of resolution [39], [40], [41], [42], [43]. Particularly, supporting previous behavioral and fMRI findings on VWM capacity revealing that VWM could hold three to four simple objects [8], [9], recent studies have consistently found that CDA amplitude was higher for four objects than for two objects when coarse information was stored [36], [37], [44]. Meanwhile, in line with the claim that VWM can hold fewer than three to four detailed objects [8], [10], the CDA difference between two and four objects vanished when detailed information was retained, regardless of whether physically complex [41], [43] or simple objects [39], [40] were retained. However, all of these studies (but [40]) manipulated the resolution of stored representations by employing different feature dimensions, such as distinct simple shapes for low resolution and physically similar random polygons for high resolution. Therefore, it is difficult to determine the entrance order of these two types of information into VWM for the same set of objects. In addition, albeit in two CDA studies, the low- and high-resolution information belonged to the same set of objects (i.e., colored random polygons) [8], [10], the participants were required to process the two types of information in separate experimental blocks, it is still impossible to determine the processing order. In the current study, to investigate the processing order between coarse and detailed information, the two types of information were derived from the same set of physically complex objects and became available to the participants at the same time, while the CDA difference between two and four objects was observed to probe the storage of the detailed information.

A critical difference exists in terms of information selection between the coarse-to-fine and all-or-none hypotheses; this enables us to examine which hypothesis captures the nature of high-resolution construction in VWM. Particularly, the coarse-to-fine hypothesis suggests that coarse information can be selected separately and stored into VWM without detailed information, while the all-or-none hypothesis predicts that coarse and detailed information are processed together. Therefore, the two hypotheses generate distinct predictions on how information is processed when the encoding and consolidation times of the memory array are manipulated and both the coarse and detailed information contained in the complex objects are available. The coarse-to-fine hypothesis predicts that the processing time of the memory array will influence the CDA difference between two and four objects by showing similar patterns as revealed in the previous studies tapping the coarse and detailed information, respectively. In contrast, the all-or-none hypothesis predicts that the participants will always select the two types of information simultaneously, regardless of processing time, and hence that there will be no difference in CDA between two and four objects.

In the following three experiments, we first conducted an experiment to select a condition in which detailed information was encoded into VWM (Experiment 1); then, on the basis of the findings of Experiment 1, Experiment 2 was conducted to test the two hypotheses by manipulating the exposure time to the memory array (100 ms vs. 500 ms). Then, Experiment 3 was conducted to further test the two hypotheses by blocking the detailed information.

## Experiment 1

In Experiment 1, we attempted to determine the condition in which detailed information was stored into VWM, since a set of new, physically complex objects were adopted as objects in the current study. To achieve this goal, we manipulated the objects’ resolution by using a within-category change (difficult comparison) condition and a cross-category change (easy comparison) condition. Previous behavioral studies indicated that for cross-category change, a distinct difference exists between the memory and test arrays, such that the processing of coarse information is sufficient to finish the task [33], [45], [46]; however, only a small difference exists for the within-category change, such that encoding the detailed information is a must except for the coarse information. To ensure that the degree of change in the cross-category condition was large enough to complete the task by encoding only the coarse information, we also added a baseline condition in which a set of distinct, simple shapes were adopted as stimuli. Previous studies suggest that only their coarse information was held in VWM [37], [41].

### Methods

#### Participants

Fourteen students (10 females, 4 males; 18–25 years old) from Zhejiang University volunteered to participate in the experiment, which employed complex objects as materials. Twelve new students (6 female, 6 male; 18–25 years old) volunteered to participate in the simple shape session. The participants reported no history of neurological problems, and all had normal or corrected-to-normal vision. All participants provided written informed consent before participating in the experiments, and all procedures were approved by the Research Ethics Board of Zhejiang University.

#### Stimuli

**Figure 1:**
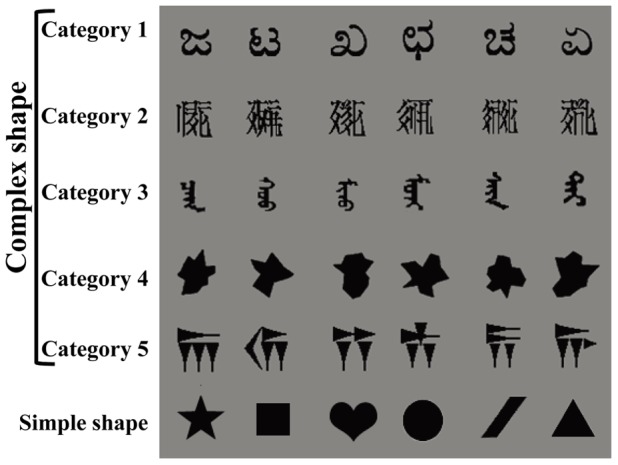
The complex and simple shapes used in Experiment 1. The stimuli in Category 4 were from ref. [8], and the other complex stimuli were new.

Five categories of physically complex, yet meaningless shapes and a group of six simple shapes were employed (Fig. 1). Each category in the complex shape condition contained six different but quite-similar shapes. Each stimulus was presented in black on a gray background and subtended about 1.23° of visual angle vertically.

#### Procedure

**Figure 2:**
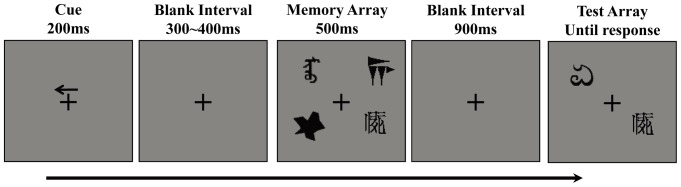
Example of a change trial in the left hemifield in Experiment 1.

The participants were seated in an electrically shielded and sound-attenuated recording chamber at a distance of 70 cm from a 17-inch monitor. The stimulus arrays were presented within two 4°×7.3° rectangular areas, centered 4° to the left and right of a central fixation cross against a gray background. The memory array were presented in the two rectangle areas, each having an equal number of 2 or 4 shapes, yet shapes and their corresponding locations in each area were selected independently (see the memory array in Fig. 2). Each time participants remembered the 2 or 4 shapes in a cued area (see details below). When the complex shapes were adopted as materials, items located within the same hemifield of the memory array were selected from five distinct categories. The positions of the memory items were randomly selected in each trial with the constraint that the center-to-center angle between items be at least 2.5°.

Each trial began with a 200-ms presentation of an arrow cue (pointing either to the left or right, each 50% of trials) above a fixation point, informing the participants which hemifield they needed to attend in this trial (see Fig. 2). After a variable delay, which ranged from 300 to 400 ms, a 500-ms memory array was displayed. Then, a 900-ms blank interval was inserted, followed by a 2000-ms presentation of the test array. To prevent the participants from making decisions on the basis of the memorized objects’ configuration information [47], we employed a partial-probe task. Particularly, we presented one shape in a location in each hemifield that was randomly selected from the ones in the memory array. The participants were required to keep their eyes fixated while remembering shapes in the cued hemifield. The test array in the cued hemifield was different from the corresponding shape in the memory array in 50% of trials; they were identical in the remaining trials. The test array in the uncued hemisphere kept constant during a trial. The participant was told to indicate whether the test item was the same as the one in that location in the memory array, with accuracy rather than response speed being stressed. When a shape changed in the cued hemifield, a new shape not used in the cued part of the memory array was randomly selected as the probe. However, in the cross-category change trials, a new category was selected, whereas in the within-category change trials, a new shape from the same category as that displayed in the corresponding location of the memory array was selected.

Each participant in the complex shape condition was tested in two blocks: within-category change and cross-category change. The display order was counterbalanced across the two blocks. The participants in the simple shape condition were tested in only one block. Each block contained 4 sessions, and each trial session lasted about 6 minutes, with a 2-minute break between blocks. Each participant performed 160 trials per set size in each change condition. Before each block, the participants completed at least 16 practice trials to make sure that they understood the instructions.

#### Behavioral analyses

Since the behavioral results are affected by many other non-storage factors, following recent CDA studies [36], [42], [43], the behavioral results section of the current study were restricted to accuracy. For estimates of VWM capacity (K), which have been generally used in previous VWM studies by using Cowan’s formula [48], please see Table S1 for detailed descriptions in the supporting information.

#### Electrophysiological recording

Electroencephalograms (EEG) were recorded from 32 scalp sites using Ag/AgCl electrodes mounted in an elastic cap. All sites were recorded with a left-mastoid reference, and the data were re-referenced offline to the algebraic average of the left and right mastoids. Vertical electrooculograms (VEOG) and horizontal electrooculograms (HEOG) were recorded with two pairs of electrodes: one pair placed above and below the left eye and another placed beside the two eyes. All inter-electrode impedance values were maintained below 5 ΚΩ. The EEG and EOG recordings employed a SynAmps amplifier (NeuroScan, Inc., Sterling, Virginia, USA) using a.05–100 Hz band pass filter, and each channel was continuously sampled at 1000 Hz for offline analysis.

### Electrophysiological Analyses

As a difference wave, CDA is constructed on the basis of a contralateral control method [49]. This method takes advantage of the contralateral organization fashion of our visual system: memories for objects presented in the right visual field are mapped onto the left hemisphere and memories for visual objects presented in the left visual field are primarily mapped onto the right hemisphere. A routine task design using the contralateral control method, as adopted in the current study, is that presenting the participants a bilateral display with equal number of objects in each hemifield, and requiring them to fix centrally and attend one hemifield according to a visual cue (e.g., the current arrow cue). In this way, the nonmnemonic processes (e.g., arousal, effort, and perceptual processing of stimuli) can be effectively controlled and a pure ERP component reflecting the information retention in VWM will be obtained by conducting a subtraction between contralateral activity and ipsilateral activity [44], [50]. Specific to the current study, we first computed the contralateral and ipsilateral waveforms. The contralateral waveforms, for instance, were computed by averaging the activity recorded at left hemisphere electrode sites when participants were cued to remember the right side of the memory array with the activity recorded from the right hemisphere electrode sites when they were cued to remember the left side. CDA was determined by subtracting the ipsilateral from the contralateral activity.

It is of note that it is critical to control the horizontal eye moment in CDA studies to ensure that the task-relevant and task-irrelevant areas of the memory display were encoded in separate hemispheres, which is the critical prerequisite of adopting the contralateral control method. Therefore, after correcting the eye blinks by using a regression procedure [51], EEG contaminated with horizontal eye movements greater than 2° (>32 µV HEOG amplitude) were excluded from analysis. Remaining artifacts (e.g., amplifier saturation, scowling, remaining eye blinks) exceeding ±75 µV in amplitude were rejected. These two EEG-rejecting criteria together resulted in 24% (SD = 11%) of trials being excluded from further analysis (an average of 244 trials were analyzed), in which eye movements resulted in most of the rejected trials. Artifact-free data were then segmented into epochs ranging from 100 ms before to 1400 ms after memory array onset for all conditions. Following our previous work [40], four pairs of electrode sites at posterior areas (P3/P4, CP3/CP4, P7/P8, and TP7/TP8) were chosen for analysis. Since the CDA result patterns were similar among these four electrodes, we pooled the data across the four electrodes, forming a representative site. The averaged CDA waveforms were smoothed by applying a low-pass filter of 10 Hz (24 dB).

Following previous studies [36], [41], [44], the present experiment adopted a measurement window of 400–1400 ms after the onset of the memory array. One-way repeated-measures ANOVAs with Set Size (two vs. four objects) as a within-subjects factor were conducted separately for each experimental block.

### Results

#### Behavioral data

**Figure 3:**
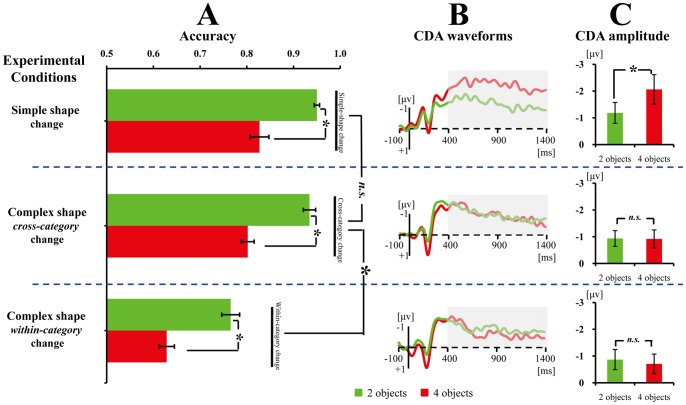
Results of Experiments 1. The mean accuracy (A), CDA waveforms (B), and averaged CDA amplitudes of the tested time window (C) for the simple shape change, cross-category change and within-category change. Error bars in Fig. 3A, and 3C denote standard error. The CDA is a difference wave, constructed by subtracting the ipsilateral from the contralateral activity according to the cued hemifield. *indicates the difference between the two conditions was significant; whereas *n.s.* indicates the difference between the two conditions was non-significant. Grey areas of the CDA waveforms denote the tested time window.

As shown in Fig. 3A, the accuracy of change detection declines as array size increased in all three conditions. Importantly, accuracy also drops as comparison difficulty rose, suggesting that comparison difficulty modulated performance. Confirming these observations, a one-way ANOVA restricted to the simple shape change condition yielded a significant main effect of Set Size, *F*(1,11) = 55.70, *p*<.001. A two-way ANOVA restricted to the complex shape group with Comparison Difficulty (cross-category change vs. within-category change) and Set Size (two vs. four objects) as within-subjects factors yielded significant main effects of Set Size, *F*(1,13) = 239.68, *p*<.001, and Comparison Difficulty, *F*(1,13) = 265.14, *p*<.001. The interaction between Comparison Difficulty and Set Size was non-significant, *F*(1,13) = .14, *p*>.2. Furthermore, a mixed ANOVA taking Shape (complex shape vs. simple shape) as a between-subjects factor and Set Size (two vs. four objects) as a within-subjects factor for the two easy comparison conditions showed that only the main effect of Set Size was significant, *F*(1,24)* = *214.08, *p*<.001; both the main effect of Shape and the interaction between the two factors were non-significant, *p*s >.2, suggesting that there was no difference in task difficulty between the simple shape change condition and cross-category change condition of the complex objects.

#### ERP data

As shown in Fig. 3B, although Set Size modulates CDA amplitude in the simple shape change condition, neither Set Size nor comparison difficulty affects CDA amplitude for the two types of complex shape change. Confirming this observation, the main effect of Set Size was significant in the simple shape change condition, *F*(1,11) = 12.92, *p*<.005, indicating that the amplitude of CDA for four objects was higher than that for two objects (see Fig. 3C). However, the difference was not significant when the analysis was restricted to either cross-category change condition, *F*(1,13) <1, or within-category change condition, *F*(1,13) <1, suggesting that there was no difference between two and four objects in terms of CDA amplitude (see Fig. 3C). Further comparisons among the four critical conditions of complex shapes did not reveal a difference between them, *p*s >.5.

In addition, Fig. 3B showed that the CDA amplitude for simple shapes was higher than that for complex shapes. We conducted a mixed ANOVA with Set Size (two vs. four objects) as a within-subjects factor and Shape (complex shape vs. simple shape) as a between-subjects factor in order to test this observation. Since there was no difference in CDA between cross-category and within-category change, we took the averaged CDA amplitude of the two conditions into statistics. The mixed ANOVA revealed a significant main effect of Set Size, *F*(1,24) = 8.96, *p*<.01, and a significant interaction between Set Size and Shape, *F*(1,24) = 13.40, *p = *.001. Post hoc contrasts showed no difference between simple and complex shapes when two objects were displayed, *p*>.1; however, the CDA amplitude was significantly more negative for simple than complex shapes when four objects were presented, *p*<.05. These results are consistent with those of a recent study [39], and support the view that the physical attributes of memorized stimuli affect CDA [40], [52].

### Discussion

Consistent with previous findings [33], [41], [43], [45], [46], both the behavioral and CDA results in the simple shape and within-category change conditions showed that the comparison difficulty of the task modulated performance and influenced the number of objects stored in VWM. However, we found a discrepancy between the behavioral and CDA results for the cross-category change condition. On the one hand, in line with previous findings [33], [45], behavioral performance in the cross-category change condition was identical to that in the simple shape condition, suggesting that the task difficulty was similar between the two conditions. Therefore, encoding coarse information was expected to be adequate to complete the task. On the other hand, the CDA results in the cross-category change condition were identical to those in the within-category change condition, implying that detailed information was also stored into VWM. Taken these results as well as previous suggestions [18], [40], [41] together, we argue that in the cross-category change condition, coarse information from three to four objects entered VWM, meanwhile, an amount of detailed information from equivalent to one or two objects was also selected into VWM.

For the following experiments, we adopted the cross-category change condition as our condition of interest. There were three reasons for selecting this condition: first, the current ERP results imply that detailed information from one to two detailed objects is encoded into VWM in this condition. Second, this selection provides the opportunity to perform a direct and critical test of the two hypotheses, as coarse information is logically sufficient to execute the cross-category change detection which is also supported by our baseline condition. Third, the large degree of the change signal in the cross-category change condition allows us to obtain relatively high performance, even under short exposure times; this would help us get enough trials for ERP analysis.

## Experiment 2

Experiment 2 had two aims. First, we intended to test a critical prediction about the impact of the encoding and consolidation times of the memory array on the two construction hypotheses. To reiterate, if construction proceeds in a coarse-to-fine manner, the encoding and consolidation times of the memory array could modulate the type of information entering into VWM, which is directly related to the resolution of the representations. Whereas the all-or-none hypothesis predicted that processing time of the memory array could only modulate the number of objects selected to enter VWM, the stored objects’ resolution would not be changed. Second, we wanted to test the coarse-to-fine explanation of Experiment 1 That is, the construction of representations (at least in the cross-category condition) proceeded in a coarse-to-fine manner and that the 500-ms processing time of the memory array was sufficient to allow for the completion of this process. If this explanation is accurate, we may observe different results from those of Experiment 1 at shorter exposure times.

We manipulated the encoding and consolidation times of the memory array by using two levels of exposure times for the memory array. While the long exposure time was still 500 ms, the short exposure time was set to 100 ms. In the 100-ms condition, it has been shown that detailed information from one to two complex objects can be extracted into VWM [43]; meanwhile, three to four low-resolution objects could be maintained [37]. If an all-or-none hypothesis is adopted in VWM, then the 100-ms condition should be adequate to encode both the coarse and detailed information from one to two objects into VWM without perceptual constraints. Consequently, the all-or-none hypothesis would predict no CDA difference between two and four objects, regardless of exposure time (100 ms vs. 500 ms). On the contrary, the coarse-to-fine hypothesis would predict that CDA may be higher for four than two objects at 100 ms exposure time, since mainly coarse information would be selected, while a replication of the results of Experiment 1 at 500 ms exposure time.

### Methods

Sixteen students (6 females, 10 males) from Zhejiang University volunteered to participate in the experiment. None of them had participated in Experiment 1. All participants provided written informed consent before participating in the experiments, and all procedures were approved by the Research Ethics Board of Zhejiang University. Two participants (one female, one male) were excluded from further analysis because of too many eye movements. The EEG and EOG were amplified by a SynAmps 2 amplifier (NeuroScan, Inc., Sterling, Virginia, USA).

**Figure 4:**
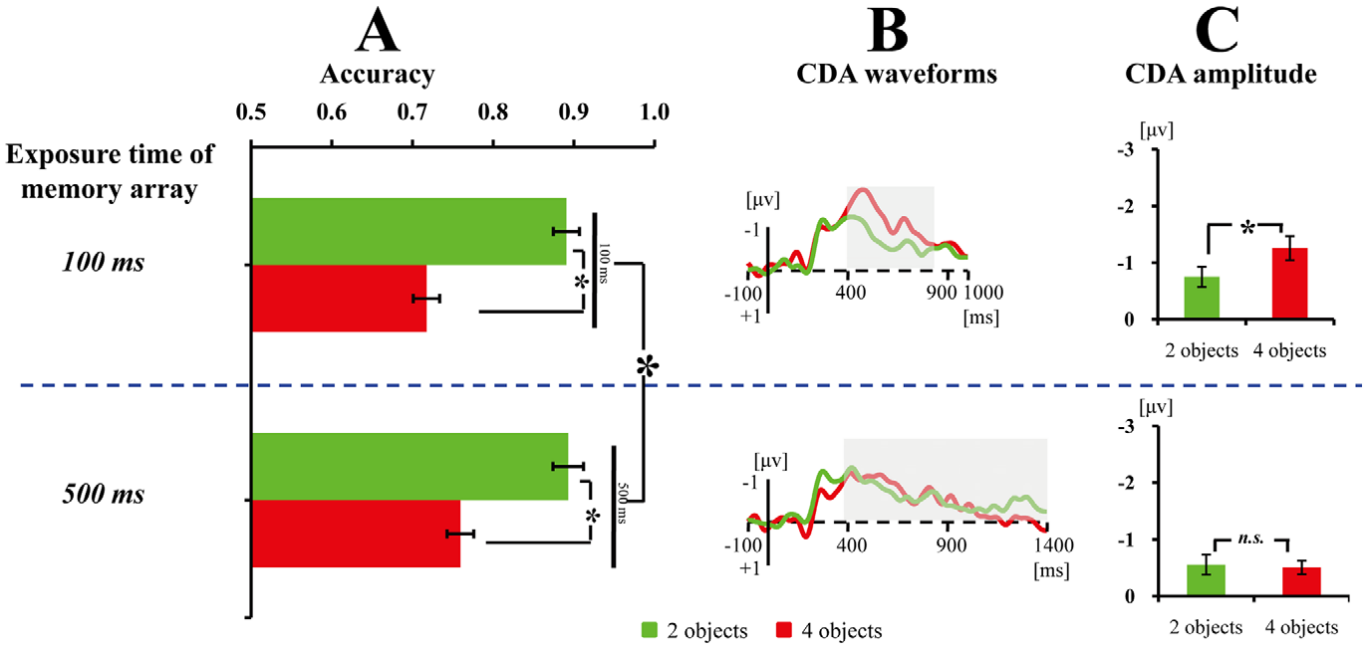
Results of Experiments 2. The mean accuracy (A), CDA waveforms (B), and averaged CDA amplitudes of the tested time window (C) for the exposure time of 100 ms and 500 ms. Error bars in Fig. 4A and 4C denote standard error. The CDA is a difference wave, constructed by subtracting the ipsilateral from the contralateral activity according to the cued hemifield. *indicates the difference between the two conditions was significant; whereas *n.s.* indicates the difference between the two conditions was non-significant. Grey areas of the CDA waveforms denote the tested time window.

Only the cross-category change condition for the complex shape in Experiment 1 was adopted. The exposure time of the memory array was either 100 ms or 500 ms, which were presented randomly. An average of 22% of trials (SD = 12%) were excluded from further analysis (249 trials left in average), eye movements resulted in most of the rejected trials. A measurement window of 400–800 ms was used for Experiment 2b, because the difference vanished completely in three out of the four examined electrode pairs after 800 ms (see Fig. 4B).

In addition, to exclude the possibility that fewer objects (*not* less information) were selected to enter VWM under 100-ms than 500-ms exposure time, we compared the CDA amplitudes among the four critical conditions (i.e., two and four objects under 100-ms and 500-ms exposure times). If this possibility is true, then the CDA amplitude in the 100-ms condition should be lower than that in 500-ms condition, since CDA amplitude is sensitive to the number of objects in VWM [36], [37], [40]. It is of note that two different measurement windows were used for the two exposure time conditions; moreover, the measurement window in the 500-ms condition contained a period of the encoding phase, yet that in the 100-ms condition only measured the maintenance phase. To make the two exposure time conditions comparable and check the number of objects retained in VWM during the maintenance phase, a new measurement window of 800–1200 ms was used for the 500-ms of condition. In this case, the CDA in both exposure time conditions started 300 ms after the offset of the stimuli and measured the same sub-period of the maintenance phase. A one-way ANOVA with Condition as a within-subjects factor was conducted on mean amplitudes. A significant main effect of Condition (*p*<0.05) was followed by post hoc Bonferroni-corrected contrasts.

### Results

#### Behavioral data

A two-way ANOVA on the mean accuracy (see Fig. 4A) was conducted with Exposure Time (100 ms vs. 500 ms) and Set Size (2 vs. 4) as within-subjects factors. The results revealed that the accuracy was higher at 500 ms (.83) than at 100 ms (.80), *F*(1,13) = 8.28, *p*<.025; the accuracy dropped as Set Size increased, *F*(1,13) = 179.18, *p*<.001. Furthermore, a significant interaction was revealed between the two factors, *F*(1,13) = 5.26, *p*<.05, suggesting that the difference between 100 ms and 500 ms was larger at set size 4 (.04) than at set size 2 (0). These results imply that more information enters VWM at 500 ms than 100 ms exposure time.

#### ERP data

Consistent with the prediction of the coarse-to-fine hypothesis, the CDA waveforms (Fig. 4B) show that exposure time to the memory array modulates the CDA difference between two and four objects. A one-way ANOVA for that time window in the 100-ms exposure condition revealed that the CDA amplitude was significantly higher for four than two objects, *F*(1,13) = 15.19, *p*<.005 (see Fig. 4C), suggesting that three to four objects are stored in VWM. In contrast, the one-way ANOVA in the 500 ms exposure time condition revealed no difference between two and four objects, *F*(1,13) <1 (see Fig. 4C), suggesting that only one to two objects are stored in VWM.

**Figure 5:**
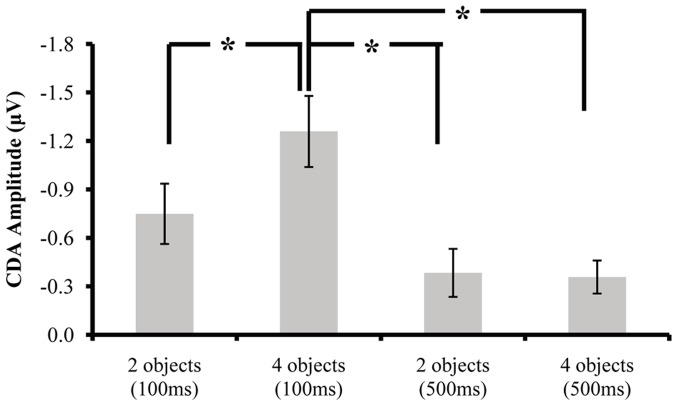
The averaged CDA amplitudes for the four critical conditions in Experiment 2. *indicates the difference between the two conditions was significant. The CDA is a difference wave, constructed by subtracting the ipsilateral from the contralateral activity according to the cued hemifield. (Error bars denote standard error).

Finally, CDA amplitudes were compared among the four critical conditions (see Fig. 5), among which the amplitude of four objects in the 100-ms condition was the highest. A one-way ANOVA revealed a significant main effect of Condition, *F*(3,39) = 18.88, *p*<.001. Post hoc contrasts showed that the CDA amplitude for four objects in the 100-ms condition was more negative than that in the other three conditions, *p*s <.025. There were no significant differences among the other three critical conditions, all *p*s >.5.

### Discussion

The behavioral results in Experiments 2 suggest that more information is encoded into VWM with an exposure time of 500 ms than 100 ms. Meanwhile, the ERP results change dramatically with the change of exposure time from 100 ms to 500 ms. The increased exposure time eliminated the CDA difference between two and four objects. Moreover, we demonstrated that the participants did not select fewer objects in 100 ms condition than in 500 ms condition, since the CDA amplitude of four objects in the 100-ms condition was the most negative one among the four critical conditions. It is also worthy noting that we replicated the above findings in one pilot experiment by adopting the two exposure times in separated blocks. On the basis of the previous CDA studies [39], [40], [41], [43], we suggest that the changed ERP pattern in the 500-ms relative to the 100-ms condition was due to the extra processing of detailed information.

In addition, the comparison results among the four critical conditions replicated our recent finding that CDA tracks the number of objects stored in VWM, regardless of resolution [40]. That is, in the 100-ms condition, CDA reflected the number of coarse objects in VWM, while in the 500-ms condition CDA tracked the number of detailed objects in VWM. This interpretation explains the discrepancy between the current behavioral (better performance in the 500-ms condition) and ERP (lower CDA amplitude for the 500-ms condition) results. The reason for the better observed performance in the 500-ms condition is that in addition to the three to four coarse objects entering VWM in the 100-ms condition, an amount of detailed information equivalent to one to two objects was also encoded in the 500-ms condition.

Therefore, we argue that the current experiment provides clear evidence supporting that the participants first encoded the coarse information, then extracted the detailed information into VWM within the trial’s exposure time (the exact timing of the encoding could not be determined from the current experiment; this issue needs to be further explored). The findings of Experiment 1 are related to the long exposure time of the memory array.

## Experiment 3

To ensure further that detailed information was processed into VWM at 500 ms exposure time (Experiments 1 and 2) and that mainly coarse information was encoded at 100 ms (Experiment 2), we directly manipulated the availability of the detailed information in Experiment 3. Specifically, we held the exposure time to memory arrays at 500 ms but blurred the objects to obscure the detailed information contained therein. If information processing proceeded in a coarse-to-fine manner in the previous two experiments, then the pattern of higher CDA for four than two objects in the 100-ms condition ofExperiment 2 should be replicated in Experiment 3.

### Methods

**Figure 6:**
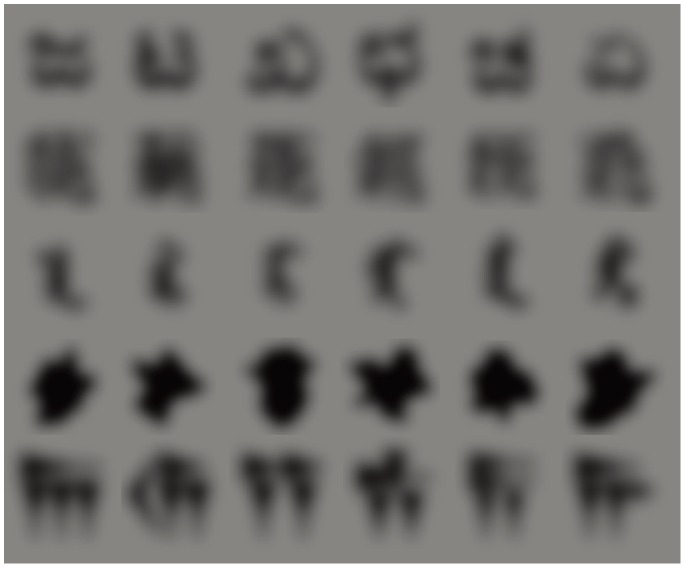
The gauss-blurred complex objects used in Experiment 3.

A group of 14 new students (6 females, 8 males) from Zhejiang University volunteered to participate in the experiment. All participants provided written informed consent before participating in the experiments, and all procedures were approved by the Research Ethics Board of Zhejiang University. A new group of stimuli was created in Photoshop by blurring the stimuli from Experiment 1 using a Gaussian filter with a 4-pixel radius; only the coarse information in these stimuli was accessible to the participants (see Fig. 6).

All aspects of Experiment 3 were identical to the cross-category change condition of Experiment 1, except that the memory arrays consisted of blurred objects. An average of 15% of trials (SD = 11%) were excluded from further analysis (272 trials left in average). A one-way repeated-measures ANOVA with Set Size (two vs. four objects) as a within-subjects factor was conducted on behavioral and ERP data, respectively.

In addition, to examine whether the current manipulation could lead to an ERP pattern as that revealed in the 100 ms condition of Experiment 2, we also compared the results between the 100 ms condition ofExperiment 2 and the current study. Mixed ANOVAs, which took Set Size (two vs. four objects) as a within-subjects factor and Experiment (Experiment 2 vs. Experiment 3) as a between-subjects factor, were conducted on behavioral and ERP data.

### Results

#### Behavioral data

**Figure 7:**
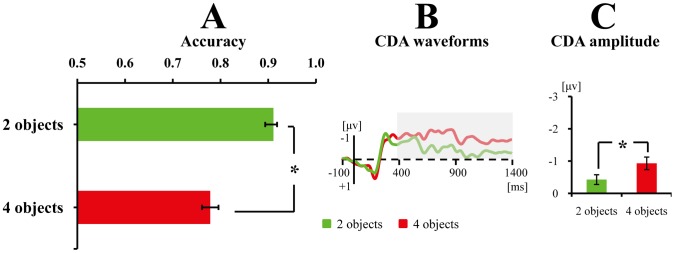
Results of Experiments 3. The mean accuracy (A), CDA waveforms (B), and averaged CDA amplitudes of the tested time window (C) for remembering the blur objects. Error bars in Fig. 7A and 7C denote standard error. The CDA is a difference wave, constructed by subtracting the ipsilateral from the contralateral activity according to the cued hemifield. *indicates the difference between the two conditions was significant; whereas *n.s.* indicates the difference between the two conditions was non-significant. Grey areas of the CDA waveforms denote the tested time window.

The one-way ANOVA showed that performance dropped as set size increased, *F*(1,13) = 93.07, *p*<.001 (see Fig. 7A).

The mixed ANOVA on accuracy of Experiments 2 (100 ms) and 3 revealed a significant main effect of Set Size, *F*(1,26) = 194.33, *p*<.001. The accuracy in Experiment 3 (.85) was significantly higher than that in the 100 ms condition ofExperiment 2 (.81), *F*(1,26) = 4.97, *p*<.05. No significant interaction was found between the two factors, *p*>0.05.

#### ERP data

Fig. 7B shows that the CDA waveforms are higher for four objects than two objects, although both were presented for 500 ms. Confirming this observation, the main effect of Set Size was significant in the one-way ANOVA, *F*(1,13) = 11.74, *p* = .005 (see Fig. 7C).

The mixed ANOVA on CDA amplitudes of Experiments 2 (100 ms) and 3 revealed a significant main effect of Set Size, *F*(1,26) = 26.53, *p*<.001. Neither the main effect of Experiment (*p*>.1) nor the Set Size × Experiment interaction (*p*>.1) reached significance.

### Discussion

Using a different manipulation, Experiment 3 replicated the CDA pattern of Experiment 2′s 100-ms condition. Hence, the current study provides direct evidence that mainly coarse information was encoded in the 100-ms condition of Experiment 2, while detailed information was also processed at the 500 ms exposure time; this supports the coarse-to-fine manner of construction in VWM.

It is of note that the behavioral performance of Experiment 3 was better than that in the 100 ms condition of Experiment 2. One possible reason is that in Experiment 3 we blurred the stimuli to make the detailed information inaccessible, yet we did not systematically manipulate the blurring parameter to make the same amount of coarse information is available to the participants between the blurred condition (Experiment 3) and the 100 ms condition (Experiment 2). It is hence possible that certain more coarse information was encoded in Experiment 3 than in the 100 ms condition of Experiment 2.

## General Discussion

The current research explored whether high-resolution representations in VWM were created in a coarse-to-fine or all-or-none manner. Three aspects of the current study enabled us to test the two construction hypotheses. First, the manipulations were performed on the same sets of objects, so that we could explore the processing order of coarse vs. detailed information while the participants formed coherent object representations. Second, the neural marker CDA allows us to online check the real information load associated with the detailed objects stored in VWM, since the storage of detailed information erases the CDA difference between two and four objects [39], [40], [41], [43]. Third, and importantly, we selected a critical point to distinguish the all-or-none hypothesis from the coarse-to-fine hypothesis: whether the coarse and detailed information enter into VWM in a simultaneous or ordered fashion. Focusing on the CDA difference between two and four objects, we found that even in the easy comparison condition, detailed information about the physically complex objects was encoded into VWM at a 500 ms exposure time, by showing no difference between two and four objects on the CDA amplitude (Experiment 1). Furthermore, this CDA difference was modulated by the exposure time to the memory array (Experiment 2) and the availability of detailed information (Experiment 3). Particularly, a distinguished CDA difference existed at 100 ms exposure time or when detailed information was not accessible; yet, this CDA-difference vanished at 500 ms exposure time. These results were consistent with findings on the construction of human faces in VWM [27], providing clear evidence for the coarse-to-fine construction of representations of common objects (as opposed to human faces) in VWM.

The current finding of coarse-to-fine processing for high-resolution representations was also supported by the results of our recent behavioral and ERP studies [53], [54], [55], [56], [57]. If coarse-to-fine processing takes place, coarse information can be encoded without accompanying detailed information, but not vice versa. In congruent with this prediction, we found that the coarse information conveyed by simple features (e.g., simple shape in Experiment 1) was selected automatically, even if it was a task-irrelevant dimension; however, detailed information could only be selected if it was task-relevant (i.e., if it was task-irrelevant, it would not be processed at all). These findings imply that the selection of coarse information has priority over the selection of detailed information.

The findings of the current study were essentially in line with the predictions of the neural object file theory [18] and a previous study on the construction of visual scenes in VWM [17], suggesting that the coarse-to-fine construction manner in perception also exists universally in VWM. Therefore, we argue that the coarse-to-fine processing order is the general manner of processing in our perceptual and VWM systems and makes our visual system work efficiently. For instance, we can use coarse information to extract a proto-object representation before processing detailed information in order to facilitate subsequent processes. Further, this manner of processing may be due to the dynamic interactions between VWM and visual perception. VWM does not passively store the final outputs of perception, but it actively engages in the different stages of online perception and stores the intermediate representations generated by those steps.

On the other hand, it is possible that “all-or-none” processing may only reflect the processing of coarse information, which is extracted quickly (or even automatically) at the beginning of VWM processing. Indeed, in a previous study, stored object information supporting all-or-none construction was essentially low-resolution information, since participants were only exposed to the simple stimuli for 100 ms, after which backward masks were displayed after a short delay (10 or 240 ms) [31]. Consistent with this possibility, a recent study found that longer exposure to a memory array allowed more information to be encoded into VWM and improved performance even for distinct colors [32].

Although the current study did not support an all-or-none hypothesis for high-resolution objects, we could not reject the “slots+averaging” model for VWM storage (of which the all-or-none encoding manner is an important component) [31]. This model claims that three to four fixed-resolution slots exist to store representations in VWM. One slot is allocated to retain each low-resolution object, while multiple slots are allocated to the same high-resolution object to accommodate its increased resolution. The current CDA results could also be explained by this model. In this sense, accuracy and CDA might reflect two different aspects of VWM: while accuracy in the easy-comparison condition (i.e., cross-category change) reflects the number of object indexes for object individuation [18] or available slots in VWM [33], [45], CDA may reflect the number of representations in VWM to which slots are allocated. In addition, supporting the existence of fixed-resolution slots in VWM, a recent study exploring resource allocation to high-resolution objects in VWM found that when the set size of memorized objects was constant, the resolution of stored objects became constant and was unaffected by the complexity of other objects [58]. Since the participants were given enough time to encode and consolidate the stimuli (i.e., 500 ms), those results do not contradict our finding that object resolution covaried with the processing time of the encoded objects before all information was accumulated into VWM.

A few limitations of the present study should be considered. First, we found that detailed information was encoded into VWM even in the easy comparison condition although its change degree was sufficiently large. This automatic extracting of the detailed information, to some extent, is reasonable since the task emphasized accuracy, extracting more detailed information into VWM improved the performance and made the participants more confident in making a response. However, the current finding was possibly context-dependent. For instance, when the blurred condition of Experiment 3 and the cross-category condition of Experiment 1 were randomly displayed in one experiment, the result pattern may be changed. Second, the current findings were based on the cross-category change, which is emphasized the processing of coarse information (or the coarse information is more important). Future work is needed to examine this issue by adopting a task (e.g., the current within-category change condition) or a type of stimuli (high-pass filtered stimuli) emphasizing the processing of high-resolution information. Finally, we adopted complex shapes as the stimuli of interest, and the coarse information consisted primarily of the objects’ shapes or contours, which can easily be recognized at a low-resolution level (e.g., Experiment 3; see also [39]). Considering that many other types of coarse and detailed information exist, future work may be needed to examine whether the current findings can be extended to other types of objects.

Beyond contributing to the understanding of the encoding mechanism of VWM, the current study also adds to the CDA literature in at least two ways. First, consistent with the study of Diamantopoulou et al. [39], in which physically simple objects were used, we found that higher CDA amplitudes were associated with remembering coarse than detailed information. This adds to accumulating evidence implying that the physical attributes of memorized stimuli indeed affect CDA amplitude [40], [41], [43], [52]. Second, by using a new method of manipulating the resolution of the representations (i.e., encoding time) and employing a different set of stimuli (i.e., shapes),Experiment 2 replicated our recent findings [40] that CDA amplitude was highest for four objects in the low-resolution condition; however, there were no CDA differences among the conditions in which two low-resolution objects, two high-resolution objects, and four high-resolution objects were presented. These results confirm our recent conclusion that CDA amplitude reflects the number of objects stored in VWM but not the resolution of the corresponding representations; however, relative differences in CDA could reflect the effect of the resolution level (at least between two and four objects). Therefore, our previous findings [40] are not constrained by a specific type of stimuli (i.e., an arrow) or a specific experimental manipulation.

### Conclusions

In summary, our findings demonstrate that the creation of high-resolution object representations in VWM proceeds in a coarse-to-fine manner.

## Supporting Information

Table S1 **Mean of hit, false alarm (FA), d’ and VWM capacity estimate (K) in all conditions.** (SC: simple shape change; CC: cross-category change; WC: within-category change).
(DOC)
