# Supplementary material for: Coarse-to-Fine Construction for High-Resolution Representation in Visual Working Memory
Source: PLoS One. 2013 Feb 28;8(2):e57913. doi: 10.1371/journal.pone.0057913 (PMC3585254; doi:10.1371/journal.pone.0057913)
Supplement: Table S1 — Mean of hit, false alarm (FA), d’ and VWM capacity estimate (K) in all conditions. (SC: simple shape change; CC: cross-category change; WC: within-category change). (DOC) [file pone.0057913.s001.doc]

**Supporting information**

We estimated the number of objects stored in VWM (see Table 1) using Cowan’s K [1]: K = S * (H - F), where K is VWM capacity, S is the number of displayed objects, H is the hit rate, and F is the false alarm rate.

In Experiment 1, mean K (remembering 4 objects) for simple shape, cross-category change of complex shape, and within-category change of complex shape was 2.52, 2.42, and 1.03, respectively.

In Experiment 2, mean K (remembering 4 objects) for 100 ms and 500 ms exposure time was 1.74 and 2.08, respectively.

In Experiment 3, mean K (remembering 4 objects) for blurred objects was 2.23.

**Table S1** Mean of hit, false alarm (FA), d’ and VWM capacity estimate (K) in all conditions. (SC: simple shape change; CC: cross-category change; WC: within-category change)

|  | **Experiment 1** | | | | | |  | **Experiment 2** | | | |  | **Experiment 3** | |
| --- | --- | --- | --- | --- | --- | --- | --- | --- | --- | --- | --- | --- | --- | --- |
|  | 2 objects | | | 4 objects | | |  | 2 objects | | 4 objects | |  | 2 objects | 4 objects |
|  | SC | CC | WC | SC | CC | WC |  | 100ms | 500ms | 100ms | 500ms |  | CC | CC` |
| Hit | .94 | .92 | .69 | .83 | .83 | .65 |  | .91 | .89 | .78 | .80 | . | .91 | .81 |
| FA | .06 | .06 | .16 | .21 | .22 | .39 |  | .13 | .11 | .35 | .28 |  | .09 | .25 |
| K | 1.77 | 1.74 | 1.06 | 2.52 | 2.42 | 1.03 |  | 1.56 | 1.57 | 1.74 | 2.08 |  | 1.64 | 2.23 |

It is of note that albeit the capacity estimates of VWM (2 - 3 objects) in simple shape and cross-category change conditions are lower than the previous estimates (i.e., 3 - 4 objects) of VWM capacity, it is close to the previous studies using 4 simple shapes to estimates the VWM capacity (e.g., [2,3]). Our pilot experiment showed that if we asked the participants to remember 6 shapes, the estimates reached to 3 - 4 objects.

**References:**

1. Cowan, N., 2001. The magical number 4 in short-term memory: A reconsideration of mental storage capacity. Behav. Brain Sci. 24, 87-114.

2. Gao, Z., Li, J., Liang, J., Chen, H., Yin, J., & Shen, M. (2009). Storing fine detailed information in visual working memory--evidence from event-related potentials. J. Vis 9, 1-12.

3. Xu, Y. (2009). Distinctive neural mechanisms supporting visual object individuation and identification. J Cogn Neurosci 21(3): 511-518.
